# The Pathology of Sunstroke

**Published:** 1876-05

**Authors:** 


The Pathology of Sunstroke.—Dr. Arndt has published a communication on this subject which is interesting, and contains much which is very suggestive. He gives a description of a march of troops on a hot day in July, 1870, with deficient shelter and no supply of water. There occurred numerous cases of sunstroke, of which no less than seven died. Post-mortem examination was only obtained in three of them, and even in these the conditions for observation were not perfect. An abnormal paleness was found in all the organs, the brain especially being as good as empty of blood. The paleness depended on emptiness of the smaller vessels and capillaries, there being an overfilling of the larger vessels in many organs, which amounted to rupture in some, so that there were ecchymoses on the pericardium, endocardium, and pleura. This overfilling of the larger vessels appears to have induced some observers to have believed that there is a hyperæmia of the organs in sunstroke; the blood, issuing from these on to the cut surface, gives an erroneous impression of congestion. At any rate, there was anæmia with swelling and œdema of the brain, liver, and kidneys. The substance of the brain was moist, and in some of the ventricles were overfilled. In the liver, kidneys, voluntary muscles, and heart there were appearances usually ascribed to parenchymatous inflammation, viz, cloudy swelling. This condition was not detected in the brain, but there it is difficult to distinguish, and, judging from the œdema and symptoms, it existed. Now, such a general cloudy swelling as this is met with in acute infective fevers, where the patients have died at the acme of the disease with high temperature. It is ascribed by some to the altered condition of the blood in these fevers; but the author believes it to be due, both there and in sunstroke, to the high temperature. The heated blood irritates the tissues, and induces this form of inflammation in the various organs involved. For there may be great alteration in the blood in these diseases, but if the temperature keeps moderately low, there seems to be little parenchymatous inflammation. Then, both in these fevers and sunstroke, the symptoms are much more related to the temperature than to the alteration of the blood. The symptoms of sunstroke are described in the various degrees of the affection. It is unnecessary to dwell on them here. There is at first a rise in temperature, with sweating, thirst, and feeling of exhaustion. These all increase in intensity if the case goes on, till a temperature of 44 C. (111 F.) may be reached. The skin is dry and burning; there is a feeling of impending death, violent palpitation and oppression in the chest, and so on. In the lesser degrees the patient may recover rapidly and completely; there is apparently no definite organic change in the organs. But in the higher degrees the brain seems to be the seat of lesions which may have a permanent effect on its functions. It is to be presumed that the nature of these lesions is inflammatory, and the fact that persons who have had sunstroke are prone to alterations of their mental condition, and even to mental diseases, is an indication of the permanency of these changes. It is to be noted that cases of infective fever, when the temperature has been very high, are said to have a similar tendency to nervous sequelæ.—*Glasgow Med. Jour.*

